# Mortality due to Cardiovascular Diseases in Women and Men in the Five Brazilian Regions, 1980-2012

**DOI:** 10.5935/abc.20160102

**Published:** 2016-08

**Authors:** Antonio de Padua Mansur, Desidério Favarato

**Affiliations:** Instituto do Coração (InCor)-HC - FMUSP, São Paulo, SP - Brazil

**Keywords:** Cardiovascular Diseases, Mortality, Epidemiology, Brazil, Stroke, Myocardial Ischemia

## Abstract

**Background:**

Studies have shown different mortalities due to cardiovascular diseases (CVD), ischemic heart disease (IHD) and cerebrovascular diseases (CbVD) in the five Brazilian regions. Socioeconomic conditions of those regions are frequently used to justify differences in mortality due to those diseases. In addition, studies have shown a reduction in the differences between the mortality rates of the five Brazilian regions.

**Objective:**

To update CVD mortality data in women and men in the five Brazilian regions.

**Methods:**

Mortality and population data were obtained from the Brazilian Institute of Geography and Statistics and Ministry of Health. Risk of death was adjusted by use of the direct method, with the 2000 world standard population as reference. We analyzed trends in mortality due to CVD, IHD and CbVD in women and men aged ≥ 30 years in the five Brazilian regions from 1980 to 2012.

**Results:**

Mortality due to: 1) CVD: showed reduction in the Northern, West-Central, Southern and Southeastern regions; increase in the Northeastern region; 2) IHD: reduction in the Southeastern and Southern regions; increase in the Northeastern region; and unchanged in the Northern and West-Central regions; 3) CbVD: reduction in the Southern, Southeastern and West-Central regions; increase in the Northeastern region; and unchanged in Northern region. There was also a convergence in mortality trends due to CVD, IHD, and CbVD in the five regions.

**Conclusion:**

The West-Central, Northern and Northeastern regions had the worst trends in CVD mortality as compared to the Southeastern and Southern regions. (Arq Bras Cardiol. 2016; [online].ahead print, PP.0-0)

## Introduction

Cardiovascular diseases (CVD) are the major cause of death in men and women in the five Brazilian geoeconomic regions.^1^ The Southeastern and Southern regions had the highest adjusted coefficients of mortality due to CVD, ischemic heart diseases (IHD) and cerebrovascular diseases (CbVD) as compared to the Northern, Northeastern and West-Central regions.^1^ Mortality due to CVD in the Southeastern and Southern regions has a pattern similar to that observed in more developed countries, where CVD have a greater participation in the population overall mortality, and mortality due to IHD is more frequent than that due to CbVD. ^2,3^ Mortality due to CVD in the Northern, Northeastern and West-Central regions has a pattern similar to that observed in developing countries, where CVD have a proportionally smaller participation in the population overall mortality, and mortality due to CbVD is more frequent than that due to IHD.^1-3^ Similarly, the reduction in mortality due to CVD, IHD and CbVD was significantly higher in the Southeastern and Southern regions as compared to that in the Northern and West-Central regions, while the Northeastern region showed an increase in mortality due to those diseases.^1,4^ Those two studies have shown an approximation of the trends in mortality due to CVD in the five regions. However, Souza et al.^1^ have assessed the mortality data due to CVD only until 2006, and Baena et al.^4^ have reported mortality data in the five regions only for IHD until 2010.

The present study aimed at assessing the trends in mortality due to CVD, IHD and CbVD, that is, if they are still maintained, in addition to updating data on mortality due to CVD in men and women in the five Brazilian regions from 1980 to 2012.

## Methods

This ecological, retrospective study based on temporal series assessed mortality due to DC, IHD and CbVD in a population aged ≥ 30 years in the five Brazilian regions (Northern, Northeastern, West-Central, Southeastern and Southern) from 1980 to 2012. Mortality data were obtained from the Brazilian Ministry of Health web portal, www.datasus.gov.br.^5^ The population data of the Brazilian Institute of Geography and Statistics (IBGE) were obtained from that same web portal. The deaths from 1990 to 1995 were classified according to the World Health Organization's International Classification of Disease (ICD), Ninth Revision (ICD-9), 1975, and adopted by the 20th World Health Assembly. According to ICD-9, diseases of the circulatory system (DCS) were encoded as 390 - 459, IHD were encoded as 410 - 414, and CbVD were encoded as 430 - 438. Mortality data from the year 1996 onwards were obtained from the Tenth Revision of ICD, and classified as follows: DCS were encoded as I00 - I99; IHD were encoded as I20 - I25; and CbVD were encoded as I60 - I69. For comparison purposes, mortality (per 100,000 inhabitants) was adjusted by using the direct standardization method, using as reference the 2000 world standard population.^6^ Simple linear regression model was used to analyze and compare mortality trends. The dependent variables were DCS, IHD and CbVD, and the independent variable was year. The significance level adopted for the statistical tests was 5% (p < 0.05). The statistical program used was SAS (SAS Institute Inc., 1989-1996, Cary, NC, USA), 9.2 version.

## Results

Overall mortality rates for men and for women due to CVD, IHD and CbVD, as well as the results of the simple linear regression analysis, are shown in Tables 1, 2, 3 and 4, respectively.

**Table 1:** Risk of death* per 100,000 inhabitants due to cardiovascular diseases (CVD), and total variation, in the total population and in men and women in the period studied (1980-2012) in the five Brazilian regions

**CVD total population**						**CVD men**						**CVD women**					
**Year**	**Northern**	**Northeastern**	**Southeastern**	**Southern**	**West- Central**	**Northern**	**Northeastern**	**Southeastern**	**Southern**	**West- Central**	**Northern**	**Northeastern**	**Southeastern**	**Southern**	**West-Central**
1980	430	261	863	791	503	464	278	986	891	529	395	245	740	691	478
1981	365	268	832	748	583	386	288	951	847	643	344	248	713	649	523
1982	329	271	789	713	538	355	295	914	827	590	302	248	664	598	485
1983	331	263	792	740	568	355	285	919	854	631	307	240	665	626	504
1984	334	276	785	727	578	362	302	916	840	641	307	250	654	615	515
1985	339	273	779	694	580	368	302	909	796	641	310	244	649	591	520
1986	341	277	749	678	565	364	304	872	790	632	319	250	625	566	497
1987	317	257	727	677	526	337	284	846	778	580	297	230	609	575	472
1988	324	276	756	711	552	349	308	889	814	612	299	244	624	607	493
1989	319	270	721	653	516	346	299	849	752	581	291	240	593	555	451
1990	324	264	700	665	483	351	296	818	767	534	298	232	582	562	431
1991	309	261	646	620	494	340	293	758	716	550	278	228	535	524	438
1992	277	263	632	615	507	310	299	743	713	569	245	227	521	517	445
1993	323	287	678	681	553	355	323	795	785	621	290	251	561	577	485
1994	327	292	663	667	579	357	325	775	766	663	296	259	552	568	495
1995	340	298	644	665	553	371	327	743	756	605	308	269	546	573	500
1996	271	266	601	593	469	296	293	702	676	520	247	239	500	510	418
1997	284	276	584	580	497	310	306	681	663	546	257	246	487	496	447
1998	294	295	576	618	500	321	331	673	714	556	267	259	479	523	444
1999	303	294	574	599	520	338	326	669	688	588	268	261	480	511	452
2000	272	277	494	532	450	308	315	583	620	511	237	240	404	444	389
2001	288	295	487	509	457	323	337	578	592	523	252	254	397	427	391
2002	283	305	483	513	482	323	345	566	596	548	243	265	401	429	415
2003	302	313	491	511	499	339	353	582	599	576	264	272	399	423	423
2004	308	332	502	523	521	355	374	595	607	597	260	291	408	439	446
2005	312	352	474	493	493	354	396	560	573	565	269	309	387	413	420
2006	329	403	492	493	504	378	454	581	572	575	279	352	403	415	433
2007	301	381	398	408	400	344	435	473	473	467	258	327	322	343	333
2008	322	383	399	396	399	369	439	474	462	465	275	327	324	329	333
2009	318	375	386	389	381	366	430	460	455	443	270	320	313	323	319
2010	298	341	387	389	384	353	396	465	456	451	242	285	310	323	316
2011	316	358	387	398	382	367	417	463	468	447	264	298	312	328	318
2012	318	357	382	381	394	369	415	458	450	455	268	299	306	313	333
var (%)	-35	27	-126	-108	-28	-26	33	-115	-98	-16	-47	18	-142	-121	-44

* adjusted by use of the direct method for the 2000 standard world population; var (%): percentage variation (2012/1980).

**Table 2:** Risk of death* per 100,000 inhabitants due to ischemic heart diseases (IHD), and total variation, in the total population and in men and women in the period studied (1980-2012) in the five Brazilian regions

**IHD total population**						**IHD men**						**IHD women**					
**Year**	**Northern**	**Northeastern**	**Southeastern**	**Southern**	**West- Central**	**Northern**	**Northeastern**	**Southeastern**	**Southern**	**West- Central**	**Northern**	**Northeastern**	**Southeastern**	**Southern**	**West- Central**
1980	91	49	267	225	106	110	60	327	278	122	72	38	207	172	90
1981	75	52	259	221	121	87	64	317	271	149	63	41	201	171	93
1982	75	54	244	205	115	94	66	303	261	141	56	43	185	150	90
1983	76	52	252	225	124	91	65	312	280	152	61	40	192	170	97
1984	71	56	247	226	121	88	70	310	285	148	55	41	185	168	95
1985	76	60	244	226	126	89	75	306	279	156	63	45	182	172	95
1986	77	61	234	212	128	90	76	291	266	158	64	45	176	159	99
1987	71	57	232	219	117	85	71	289	273	143	56	43	175	165	91
1988	73	61	239	228	123	88	76	301	282	151	58	46	177	174	94
1989	69	60	228	204	116	82	74	287	252	139	56	47	170	155	93
1990	77	60	218	206	106	95	74	273	257	132	60	45	162	155	80
1991	74	61	202	198	114	91	76	252	246	137	58	46	151	150	91
1992	64	61	191	194	118	80	75	241	242	147	48	47	141	146	89
1993	74	65	199	211	126	91	80	251	262	157	58	50	148	160	95
1994	73	66	197	209	127	91	81	247	259	158	56	51	148	160	95
1995	79	71	194	210	127	92	85	239	258	150	66	56	149	162	10
1996	66	66	186	194	118	79	79	234	239	142	52	52	138	148	93
1997	68	70	181	188	118	82	84	227	229	142	54	55	135	147	94
1998	68	73	179	202	122	81	89	225	248	150	55	57	133	156	95
1999	74	73	180	201	124	88	87	227	247	155	60	59	134	156	93
2000	65	71	157	180	115	80	87	200	224	143	50	55	113	136	87
2001	67	78	155	171	120	82	96	198	213	150	52	60	113	128	89
2002	66	82	156	173	129	84	100	198	216	159	48	64	114	129	99
2003	72	84	158	169	134	88	103	202	213	168	56	66	114	125	99
2004	78	90	161	173	143	99	109	206	216	179	56	71	116	131	10
2005	75	94	150	163	134	94	115	192	204	169	56	74	108	121	99
2006	81	109	156	162	139	104	133	200	205	172	59	86	111	120	10
2007	78	106	126	135	111	97	130	164	171	142	58	82	89	98	80
2008	82	109	127	129	113	104	134	165	164	145	61	85	89	93	81
2009	86	108	122	126	110	110	133	159	162	142	62	83	85	90	78
2010	81	103	124	125	112	105	128	161	159	147	57	77	86	92	77
2011	85	109	125	128	115	110	137	162	165	148	61	82	87	91	82
2012	84	111	125	121	121	118	137	163	158	157	50	85	86	85	86
var (%)	-8	56	-114	-86	12	7	128	-101	-76	22	-44	55	-140	-102	-5

* adjusted by use of the direct method for the 2000 standard world population; var (%): percentage variation (2012/1980).

**Table 3:** Risk of death* per 100,000 inhabitants due to cerebrovascular diseases (CbVD), and total variation, in the total population and in men and women in the period studied (1980-2012) in the five Brazilian regions

**CbVD total population**						**CbVD men**						**CbVD women**					
**Year**	**Northern**	**Northeastern**	**Southeastern**	**Southern**	**West- Central**	**Northern**	**Northeastern**	**Southeastern**	**Southern**	**West- Central**	**Northern**	**Northeastern**	**Southeastern**	**Southern**	**West- Central**
1980	117	82	275	207	110	121	82	303	225	114	113	82	247	188	107
1981	96	88	282	213	146	97	89	312	234	157	95	88	252	193	134
1982	98	87	270	207	137	98	89	302	235	147	99	85	238	178	128
1983	98	83	266	212	148	97	84	300	239	163	99	83	232	185	133
1984	103	89	276	209	151	104	92	312	232	165	102	86	240	186	137
1985	103	86	271	201	148	105	89	309	226	159	101	83	233	176	137
1986	102	89	266	202	148	103	93	303	234	160	100	86	229	170	135
1987	105	86	254	201	138	107	90	289	227	148	103	82	220	174	128
1988	107	93	265	208	146	112	98	304	234	162	103	89	226	182	131
1989	102	91	251	199	144	107	97	289	226	155	97	86	212	172	134
1990	101	89	249	203	135	104	95	288	231	149	97	82	209	176	122
1991	99	87	229	192	132	105	93	266	220	147	92	81	192	164	118
1992	84	90	229	189	136	90	98	266	217	148	79	81	192	160	125
1993	104	97	246	204	158	108	105	286	231	175	101	88	206	176	141
1994	106	97	239	199	168	112	104	276	226	190	100	90	202	172	146
1995	111	97	233	198	148	119	104	268	224	163	103	90	198	173	133
1996	89	81	155	164	121	93	87	179	183	135	84	76	130	144	107
1997	92	85	152	165	131	94	91	177	190	146	89	79	128	140	116
1998	97	89	147	176	133	103	97	171	205	150	90	81	122	146	117
1999	93	88	141	167	141	102	94	165	192	158	85	81	118	142	125
2000	89	82	121	144	113	95	91	142	167	129	83	73	101	120	98
2001	93	91	119	141	114	101	101	142	162	129	84	81	97	119	99
2002	93	92	119	140	119	102	103	139	163	134	83	82	99	118	103
2003	98	94	118	139	121	105	104	140	162	138	92	84	96	116	105
2004	97	97	116	142	124	109	106	137	164	142	85	88	96	121	105
2005	101	103	109	134	114	111	113	127	153	124	90	93	91	114	104
2006	109	120	115	135	119	119	131	134	155	134	98	108	96	115	105
2007	96	109	91	111	89	105	122	107	127	102	87	95	75	95	77
2008	105	108	89	106	92	115	121	105	124	106	95	94	74	88	78
2009	100	103	87	105	85	110	115	103	121	96	91	91	72	90	74
2010	91	94	86	107	89	102	105	103	125	102	80	82	69	88	75
2011	95	97	84	106	84	107	112	100	123	97	82	82	68	88	71
2012	93	97	81	103	86	104	111	96	120	98	82	82	66	86	74
var (%)	-26	15	-240	-101	-28	-16	26	-216	-88	-16	-38	0	-274	-119	-45

* adjusted by use of the direct method for the 2000 standard world population; var (%): percentage variation (2012/1980).

**Table 4:** Simple linear regression model for mortality due to cardiovascular diseases (CVD), ischemic heart diseases (IHD) and cerebrovascular diseases (CbVD) in men and women in the period studied (1980-2012) in the five Brazilian regions

	**Total**				**Men**				**Women**			
	**Raj^2^**	**β**	**95%CI**	**P**	**Raj^2^**	**β**	**95%CI**	**P**	**Raj^2^**	**β**	**95%CI**	**P**
CVD Northern	0.23	-1.56	-2.53 – -0.59	0.003	0.02	-0.68	-1.78 – 0.42	0.220	0.51	-2.45	-3.30 – -1.60	< 0.0001
CVD Northeastern	0.70	3.72	2.85 – 4.60	< 0.0001	0.76	4.75	3.89 – 5.82	< 0.0001	0.57	2.58	1.78 – 3.39	< 0.0001
CVD Southeastern	0.97	-15.30	-16.18 – -14.43	< 0.0001	0.97	-17.22	-18.23 – -16.20	< 0.0001	0.97	-13.38	-14.17 – -12.60	< 0.0001
CVD Southern	0.93	-12.12	-13.32 – -10.92	< 0.0001	0.93	-13.53	-14.84 – -12.21	< 0.0001	0.92	-10.70	-11.81 – -9.57	< 0.0001
CVD West-Central	0.63	-5.17	-6.59 – -3.76	< 0.0001	0.50	-4.70	-6.37 – -3.03	< 0.0001	0.74	-5.64	-6.86 – -4.44	< 0.0001
IHD Northern	0.14	0.14	-0.10 – 0.38	0.238	0.16	0.45	0.11 – 0.80	0.012	0.07	-0.17	-0.35 – 0.02	0.072
IHD Northeastern	0.90	1.97	1.73 – 2.22	< 0.0001	0.88	2.41	2.10 – 2.73	< 0.0001	0.90	1.54	1.36 – 1.73	< 0.0001
IHD Southeastern	0.97	-4.64	-4.92 – -4.36	< 0.0001	0.97	-5.47	-5.83 – -5.12	< 0.0001	0.97	-3.81	-4.03 – -3.60	< 0.0001
IHD Southern	0.85	-3.27	-3.76 – -2.78	< 0.0001	0.87	-3.92	-4.47 – -3.37	< 0.0001	0.82	-2.62	-3.10 – -2.17	< 0.0001
IHD West-Central	0.48	0.11	-0.21 – 0.44	0.479	0.10	0.43	0.02 – 0.84	0.023	0.06	-0.76	-1.63 – 0.12	0.089
CbVD Northern	0.08	-0.24	-0.49 – 0.01	0.056	0.01	0.11	-0.17 – -0.39	0.440	0.45	-0.60	-0.84 – -0.37	< 0.0001
CbVD Northeastern	0.38	0.56	0.31 – 0.81	< 0.0001	0.62	0.95	0.68 – 1.22	< 0.0001	0.01	0.14	-0.10 – 0.38	0.234
CbVD Southeastern	0.92	-7.51	-8.29 – -6.74	< 0.0001	0.91	-8.27	-9.20 – -7.34	< 0.0001	0.94	-6.74	-7.37 – -6.11	< 0.0001
CbVD Southern	0.90	-3.84	-4.38 – -3.40	< 0.0001	0.88	-4.13	-4.68 – -3.59	< 0.0001	0.92	-3.56	-3.94 – -3.18	< 0.0001
CbVD West-Central	0.56	-1.81	-2.38 – -1.24	< 0.0001	0.45	-1.72	-2.39 – -1.05	< 0.0001	0.67	-1.91	-2.39 – -1.43	< 0.0001

95% CI: 95% confidence interval.

**Figure 1:**
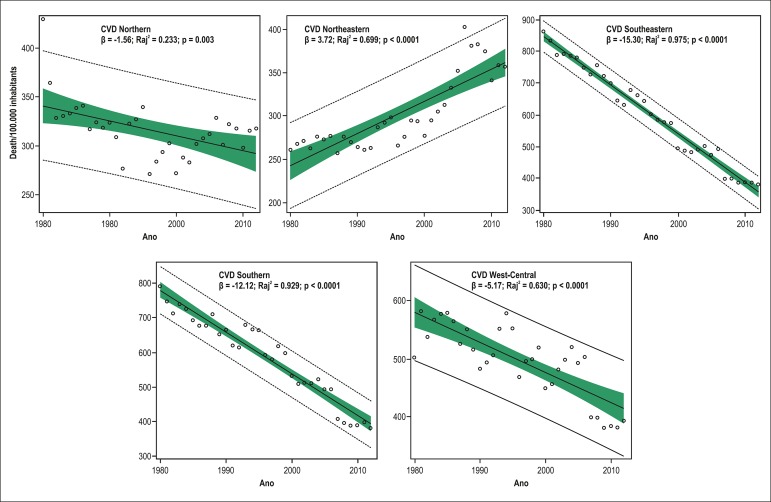
Simple linear regression analysis of mortality due to cardiovascular diseases (CVD) in individuals aged ≥ 30 years in the five Brazilian regions from 1980 to 2012.

Mortality due to CVD increased in the Northeastern region from 1980 to 2012, as follows: 27% in the total population, 33% in men, and 18% in women. In the other regions, a reduction in mortality was observed in the total population, in men and in women. The reductions were more significant in the Southern and Southeastern regions, being greater than 95% in mortality from 1980 to 2012 (Table 1, Figure 1).

**Figure 2:**
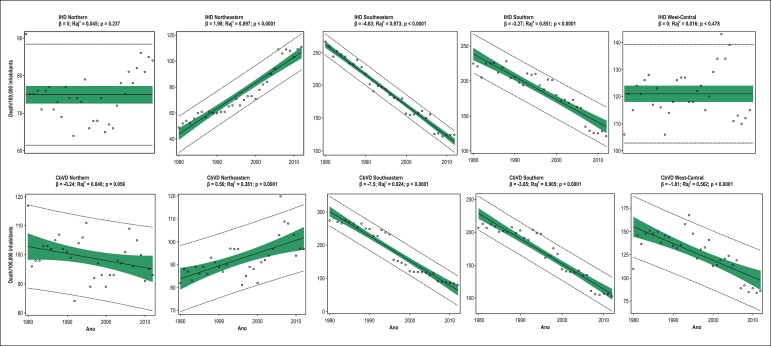
Simple linear regression analysis of mortality due to ischemic heart diseases (IHD) and cerebrovascular diseases (CbVD) in individuals aged ≥ 30 years in the five Brazilian regions from 1980 to 2012.

**Figure 3:**
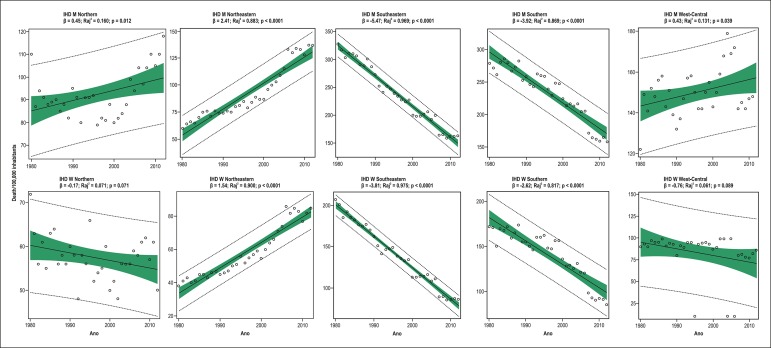
Simple linear regression analysis of mortality due to ischemic heart diseases (IHD) in men (M) and women (W) aged ≥ 30 years in the five Brazilian regions from 1980 to 2012.

The simple linear regression analysis showed: from 1980 to 2012, mortality due to IHD remained unaltered in the Northern (β = 0.02; R_aj_^2^ = 0.045; p = 0.237) and West-Central (β = 0.01; R_aj_^2^ = 0.016; p = 0.478) regions; increased in the Northeastern region (β = 1.98; R_aj_^2^ = 0.897; p<0.0001); and decreased in the Southeastern (β = -4.63; R_aj_^2^ = 0.973; p < 0.0001) and Southern (β = -3.27; R_aj_^2^ = 0.851; p < 0.0001) regions (Tables 2 and 4; Figure 2). In men, mortality due to IHD increased in the Northern (β = 0.45; R_aj_^2^ = 0.160; p = 0.012), Northeastern [ β = 2.41 (95%CI: 2.10-2.75); R_aj_^2^ = 0.883; p < 0.0001] and West-Central (β = 0.43; R_aj_^2^ = 0.131; p = 0.039) regions. The most important increase occurred in the Northeastern region (128%), followed by the West-Central (22%), and Northern (7%) regions (Tables 2 and 4, Figure 3). In women, mortality due to IHD increased in the Northeastern region (β = 1.54; R_aj_^2^ = 0.900; p < 0.0001), and remained unaltered, but with a reduction trend, in the Northern (β = -0.17; R_aj_^2^ = 0.071; p = 0.071) and West-Central (β = -0.76; R_aj_^2^ = 0.061; p = 0.089) regions. The Northeastern region had the greatest increase in mortality due to IHD (55%) (Tables 2 and 4, Figure 3).

**Figure 4:**
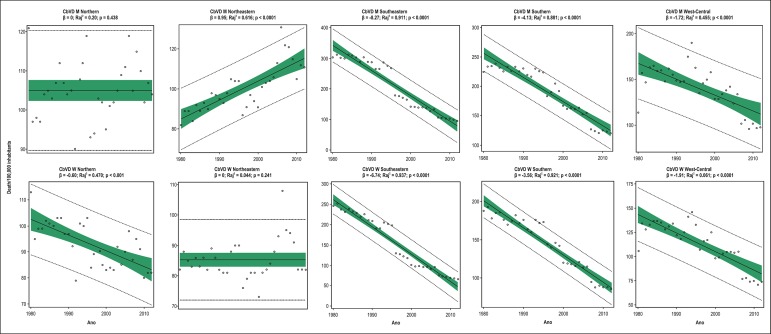
Simple linear regression analysis of mortality due to cerebrovascular diseases (CbVD) in men (M) and women (W) aged ≥30 years in the five Brazilian regions from 1980 to 2012.

**Figure 5:**
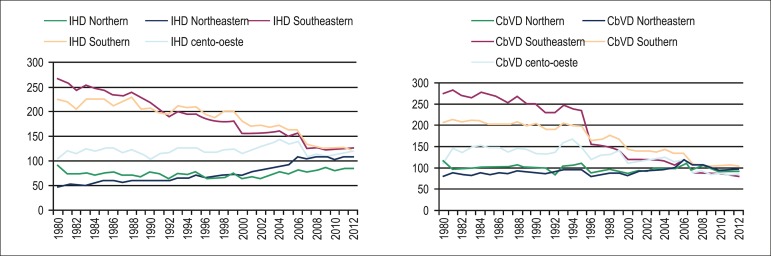
Convergence of trends in mortality due to ischemic heart diseases (IHD) and cerebrovascular diseases (CbVD) in the five Brazilian regions from 1980 to 2012.

Simple linear regression analysis showed that, from 1980 to 2012, mortality due to CbVD remained unaltered, but with a reduction trend, in the Northern region (β = -0.24; R_aj_^2^ = 0.840; p = 0.056), increased in the Northeastern region (β = 0.56; R_aj_^2^ = 0.381; p < 0.0001), and had a significant reduction in the Southeastern (β = -7.5; R_aj_^2^ = 0.924; p < 0.0001), Southern (β = -3.85; R_aj_^2^=0.905; p < 0.0001) and West-Central (β = -1,81; R_aj_^2^ = 0,562; p < 0,00) regions. Mortality due to CbVD increased in the Northeastern region by 15%, while significant reductions of 240% and 101% occurred in the Southeastern and Southern regions, respectively (Tables 3 and 4, Figure 2). In men, mortality due to CbVD increased in the Northeastern region (β = 0.95; R_aj_^2^ = 0.616; p < 0.0001), remained unaltered in the Northern region (β = 0; R_aj_^2^ = 0.020; p = 0.438), and decreased in the Southeastern (β = -8.27; R_aj_^2^ = 0.911; p < 0.0001), Southern (β = -4.13; R_aj_^2^ = 0.881; p < 0.0001) and West-Central (β = -1.72; R_aj_^2^ = 0.455; p < 0.0001) regions. In men, mortality due to CbVD increased in the Northeastern region by 26%, the most significant reductions of 216% and 88% occurring in the Southeastern and Southern regions, respectively (Tables 3 and 4, Figure 4). In women, mortality due to CbVD remained unaltered in the Northeastern region (β = 0; R_aj_^2^ = 0.044; p = 0.241), and decreased in the Northern (β = -0.60; R_aj_^2^ = 0.470; p<0.001), Southeastern (β = -6.74; R_aj_^2^ = 0.937; p < 0.0001), Southern (β = -3.56; R_aj_^2^ = 0.921; p < 0.0001) and West-Central (β = -1.91; R_aj_^2^ = 0.061; p < 0.0001) regions. In women, the reduction in mortality due to CbVD was more important in the Southeastern and Southern regions, 274% and 119%, respectively (Tables 3 and 4, Figure 4). The convergence of the trends in mortality due to IHD and CbVD observed in the five Brazilian regions resulted mainly from the reduction in mortality due to those diseases in the Southeastern and Southern regions. The convergence of mortality due to CbVD was significant from 1997 onwards, while, for IHD, that occurred only from 2007 onwards (Figure 5).

## Discussion

This study showed the highest reductions in mortality due to CVD, IHD and CbVD in the Southeastern and Southern regions, while the Northeastern region had mortality due to those diseases increased. The results varied in the Northern and West-Central regions. Therefore, the Southeastern and Southern regions behaved similarly to the most developed countries, with a persistent trend of reduction in mortality due to CVD.^7,8^

On the other hand, the mortality trends of the other regions behaved similarly to those of developing countries. The population's more limited access to a more appropriate health care system, in addition to socioeconomic and cultural aspects, might justify those trends. For example, the control of risk factors accounted for at least a 50% reduction in mortality due to CVD in more developed countries.^9^

A recent report of the 2013 Brazilian National Health Research ( *Pesquisa Nacional de Saúde* - PNS) showed better performance of the Southeastern and Southern regions regarding the diagnosis and treatment of the major risk factors for CVD.^10^ The PNS data showed a higher consumption of fruits and vegetables and greater practice of physical activity in the Southeastern and Southern regions. Regarding risk factors [systemic arterial hypertension (SAH), dyslipidemia and diabetes], the Southeastern and Southern regions showed: greater proportion of individuals aged ≥ 18 years measuring blood pressure; higher use of anti-hypertensive drugs; greater access to at least one medication obtained from the Popular Pharmacy Program; and more frequent measurement of serum glucose, total cholesterol and triglyceride levels.^10^ Briefly, the population's access to the health care system was better in the Southeastern and Southern regions.

Similarly, regarding risk factor assessment, that PNS report showed that women performed better as compared to men, which can even intensify the already existing natural protection of women against the atherosclerotic process, and, thus, against cardiovascular events.

In addition, the better access to the health care system in the Southeastern and Southern regions can justify the greater reduction in mortality due to CbVD as compared to IHD. That results from the fact that the logistics involved in the diagnosis and treatment of SAH, the major risk factor for CbVD, is significantly less complex than that required for IHD. Ischemic heart diseases involve more risk factors, such as dyslipidemia, smoking habit, diabetes and SAH, and their diagnosis depend on more complex complementary tests.

In addition to the drug treatment complexity, there is limited availability of the intervention treatment, restricted to large urban centers. Such diagnostic and therapeutic limitations can justify the heterogeneity in the risk of death due to acute myocardial infarction in the different Brazilian regions.^11^

Similarly, social inequalities and low educational level are additional conditions associated with higher mortality due to CVD.^12-14^ The Southern and Southeastern regions have the highest urban developing indices, which is assessed by the progress of the regions in three basic dimensions: income, educational level and health.^15,16^ Half of the mortality due to CVD before the age of 65 years can be attributed to poverty.^13^ Similarly, the educational level has an inverse relationship with mortality due to CVD. Inadequate feeding, insufficient physical activity, alcohol consumption and smoking are important risk factors for DVC and more prevalent in the least favored social levels.^17^ Therefore, primary and secondary prevention programs aimed at those population strata can significantly impact morbidity and mortality due to CVD. For example, the "Family Health Strategy" program facilitated actions for health promotion and perfected the process of prevention and early diagnosis of the major risk factors for CVD.^18^

Another important point observed in our study was the convergence of the trends in mortality due to IHD and CbVD in the Brazilian regions. The convergence of the trends in mortality due to IHD occurred from 2007 onwards, while that due to CbVD occurred 10 years earlier. That behavior reflects in the earlier and steepest drop in mortality due to CbVD, resulting in the epidemiological transition phenomenon, which is predominance of mortality due to IHD over that due to CbVD.^19^

This study's major limitations relates to the quality of Brazilian mortality data, such as errors related to the diagnosis and accuracy of death certificates, ill-defined causes of deaths and data inputting errors. The number of death certificates with symptoms, signs and ill-defined health conditions reported as cause of death is an indirect indicator of the data quality pattern. Despite the progressive improvement, the number of death certificates with those characteristics in the Northeastern, Northern and West-Central regions is still significant.^20,21^

In addition, validation studies for mortality rate data are not available in most Brazilian states or cities. Thus, the reduction in the number of death certificates with symptoms, signs and ill-defined health conditions reported as cause of death can redirect to the increase in the number of death certificates due to CVD, and consequently, artificially reflect as an increase in mortality due to CVD in the Northeastern, Northern and West-Central regions.

## Conclusion

The persistence of those mortality trends in the five Brazilian regions will lead, in a few years, to an inversion in the risk of death in the regions, making the Northeastern region, and to a lesser extent, the Northern and West-Central regions, those with the highest coefficients of mortality due to CVD. Thus, intensification of preventive public health policies for CVD and improvement in socioeconomic conditions, especially in the Northeastern region, might result in similar coefficients of mortality in the five Brazilian regions.
